# Endophthalmitis at a tertiary referral center: Characteristics and treatment outcomes over three decades

**DOI:** 10.3389/fcell.2022.952375

**Published:** 2022-08-08

**Authors:** Wen-Fei Zhang, Xin-Yu Zhao, Li-Hui Meng, Huan Chen, You-Xin Chen

**Affiliations:** ^1^ Department of Ophthalmology, Peking Union Medical College Hospital, Chinese Academy of Medical Sciences, Beijing, China; ^2^ Key Laboratory of Ocular Fundus Diseases, Chinese Academy of Medical Sciences & Peking Union Medical College, Beijing, China

**Keywords:** endophthalmitis, pars plana vitrectomy, pathogens, retinal detachment, antibiotics

## Abstract

**Purpose:** To explore the incidence, pathogens, treatment, and prognosis of endophthalmitis.

**Methods:** Patients who were diagnosed with endophthalmitis from January 1990 to October 2020 at Peking Union Medical College Hospital were retrospectively reviewed and examined. Subgroup analysis was conducted regarding different initial treatment methods for eyes without concurrent retinal detachment (RD) at presentation.

**Results:** A total of 249 eyes of 233 patients were included in this retrospective study. The most common clinical scenario was exogenous endophthalmitis (60.6%). The most frequent bacteria and fungi were coagulase-negative staphylococci (10.0%) and candida (6.8%), respectively. Retinal with/without choroid detachment was the most common complication after treatment. Patients with endogenous endophthalmitis were more likely to have binocular involvement; there were also more patients with diabetes mellitus or immunosuppressive diseases, and the prognosis of visual acuity (VA) was poorer. There were more eyes with concurrent RD at presentation that underwent serious complications after treatment (*p* < 0.05), and the visual outcome was worse than that without concurrent RD (*p* < 0.05). Subgroup analysis was conducted according to different initial treatments in eyes without concurrent RD. Group 1 received pars plana vitrectomy (PPV) with intravitreal injection of antibiotics (IVI) as initial treatment, Group 2 was initially treated with IVI only, and Group 3 was initially treated with nonsurgical treatment. More eyes that initially received IVI alone and nonsurgical treatment required additional treatments, especially additional PPV. VA in both Groups 1 and 2 significantly improved by the final VA. However, there was no significant difference in final VA between the two groups. There was an insignificant trend that serious posttreatment complications were more common in Group 1. In Group 1, 17 eyes received silicone oil or gas tamponade at the same time, whereas 62 did not. Eyes that were initially treated with PPV + IVI while without tamponade needed more additional treatments and additional IVI.

**Conclusion:** Endophthalmitis is a devastating intraocular disease and requires early intervention. Endogenous endophthalmitis has a poorer visual prognosis than exogenous entity. PPV + IVI as an initial treatment may reduce additional therapy.

## Introduction

Endophthalmitis, which often refers to bacterial or fungal infection of the vitreous and/or aqueous, is one of the most devastating intraocular diseases and usually causes irreversible visual impairment. It can be endogenous, caused by hematogenous bacteriaemia and/or fungaemic spreading to the eye, or exogenous, arising after trauma or eye surgery (Durand, 2013; Durand, 2017). In the past few decades, the main causes, pathogens, and treatments of endophthalmitis have changed significantly. Although the incidence of endophthalmitis varies among countries and times, exogenous endophthalmitis is the most important cause, accounting for 92–98% of total endophthalmitis, and mostly correlates with cataract surgery. By contrast, endogenous endophthalmitis is relatively rare (Jackson et al., 2014; Durand, 2017; Spelta et al., 2021). Since the approval of antivascular endothelial growth factor (anti-VEGF) drugs, endophthalmitis caused by intraocular injection has increased rapidly and is even more common than another postoperative endophthalmitis (Simunovic et al., 2012).

The treatment of endophthalmitis, especially at the time of vitrectomy, is always under discussion. More than 20 years ago, the Endophthalmitis Vitrectomy Study (EVS) compared the efficacy of the vitrectomy group with the inject-only group and concluded that vitrectomy was beneficial only for eyes with light perception (LP) or worse than LP (Endophthalmitis Vitrectomy Study Group, 1995). With the increasing understanding of the pathogenesis of endophthalmitis and advances in surgical techniques, some view the EVS protocol as outdated and suggest that aggressive vitrectomy could achieve a better visual outcome. Advocates point out that vitrectomy enables sample collection for analysis and better antibiotic distribution and reduces microbial load, toxins, and inflammatory factors (Kuhn and Gini, 2005; Kuhn and Gini, 2006). Critics believe that surgery may lead to severe complications such as retinal tears and retinal detachment (RD) and a faster turnover of antibiotics in the vitreous. At the same time, immediate surgical treatment is unrealistic and unattainable in most clinical scenarios. Even large vitreoretinal units may have difficulty in allocating operating room capacity within such tight time frames (Maguire, 2008; Clarke et al., 2018). These suggest that there is a lack of consensus on better treatment at the initial presentation for endophthalmitis patients. As a result, the debate over early vitrectomy has continued for decades (Kuhn and Gini, 2005; Kitsche et al., 2020).

The purpose of our study was to explore the incidence, pathogens, treatment, and prognosis of endophthalmitis. The clinical characteristics of endophthalmitis with/without concurrent RD at presentation were evaluated and the prognosis of different initial treatment methods for eyes without concurrent RD was discussed, to provide a reference for ophthalmologists.

## Materials and methods

This was a single-center retrospective study. We reviewed all patients with endophthalmitis admitted to Peking Union Medical College Hospital from January 1990 to October 2020 and recorded the demographic characteristics, general and ocular history, clinical settings, microbiological results, initial treatment, additional treatments, initial and final best-corrected visual acuity (BCVA), and serious complications after treatment. This study adhered to the Declaration of Helsinki of the World Medical Association and was approved by the Institutional Review Board/Ethics Committee of Peking Union Medical College Hospital. Written informed consent was provided to each patient before the surgery. The clinical diagnosis of endophthalmitis was determined using clinical signs and symptoms, including eye pain, the loss of vision, eyelid edema, conjunctival congestion and chemosis, anterior chamber inflammation (flare, cells, hypopyon, pupillary fibrin membrane, and blebitis), vitritis, decreased red reflex, and vitreous opacity in B-scan ultrasound (Foster et al., 1994). Treatment of endophthalmitis was determined by individual physicians. Initial treatments included pars plana vitrectomy (PPV) together with an intravitreal injection of antibiotics (IVI) (with/without tamponade), IVI only, and nonsurgical treatments (including a subconjunctival depot with antibiotics along with a local and systemic antibiotic therapy). The additional treatment referred to postinitial interventions to control endophthalmitis or to treat serious complications. Silicone oil removal was not included. In addition, all patients underwent anterior chamber or vitreous sampling for microbiological investigation prior to operation in our hospital. The BCVA was converted to the logarithm of the minimum angle of resolution (LogMAR) equivalents. LogMAR 3.0, 2.7, 2.3, and 1.9 were equal to no LP (NLP), LP, hand motion, and counting fingers, respectively.

All data were collected and evaluated by two retinal specialists (W.-F.Z. and X.-Y.Z.). The mean ± SD was used to evaluate continuous variables. Counts and percentages were used to categorize variables. Continuous variables were analyzed using an independent *t*-test, a paired *t*-test, or a nonparametric test (if not normal distribution). The chi-square test or Fisher’s exact test was used to analyze categorical variables. Multivariate logistic regression analysis was used to identify factors associated with poor visual prognosis. SPSS 26.0 (SPSS Inc., Chicago, Illinois, United States) was used for statistical analysis; *p* < 0.05 was considered statistically significant.

## Results

### General data

A total of 261 patients (277 eyes) were recorded with endophthalmitis. Enucleation or enucleation was performed in 28 eyes of 28 patients at presentation. At last, 249 eyes of 233 patients were included in this study (Table 1).

**TABLE 1:** Baseline demographics and clinical characteristics of patients with endophthalmitis.

Number of patients and eyes	General data (*n* = 249 eyes of 233 Patients)
Sex(male), n(%)	140 (60.1)
Age, years	47.20 ± 20.48
Side(R), eyes(%)	108 (46.4)
Presence of DM, n(%)	46 (19.7)
Presence of HTN, n(%)	50 (21.5)
Immunosuppression, n(%)	16 (6.9)
Initial logMAR BCVA	2.22 ± 0.61
Ocular history, eyes(%)	51 (20.5)
Glaucoma	22 (8.8)
RVO	11 (4.4)
PDR	7 (2.8)
AMD	4 (1.6)
Idiopathic choroidal neovascularization	4 (1.6)
Thyroid eye disease	1 (0.4)
Coloboma of choroid and iris	1 (0.4)
Corneal endothelial decompensation	1 (0.4)
Clinical scenarios, eyes(%)	
Endogenous	98 (39.4)
Perforating injuries	62 (24.9)
Post-cataract surgery	43 (17.3)
Post-intravitreal injection	18 (7.2)
Post-glaucoma surgery	12 (4.8)
Post-vitrectomy	11 (4.4)
Post-scleral buckling	5 (2.0)
Pathogens, eyes(%)	
Coagulase-negative staphylococci	25 (10.0)
Streptococcus	6 (2.4)
Staphylococcus aureus	6 (2.4)
Enterococcus faecalis	5 (2.0)
Gram-negative bacilli	14 (5.6)
Gram-positive bacilli	8 (3.2)
Candida	17 (6.8)
Aspergillus	8 (3.2)
Other fungi	7 (2.8)
Negative	101 (40.6)
NA	52 (20.9)
Serious complications after treatment, eyes(%)	99 (39.8)
Retinal with/without choroid detachment	53 (21.3)
Evisceration or enucleation	20 (8.0)
Decreased VA to NLP	23 (9.2)
Corneal decompensation	3 (1.2)
Final logMAR BCVA	1.97 ± 0.90
Time of follow up (days)	326.18 ± 684.66

Other fungi included Paecilomyces lilacinus, Cladosporium, Fusarium, Paecilomyces, Mucor, and Cryptococcus.

AMD, age-related macular degeneration; BCVA, best-corrected visual acuity; DM, diabetes mellitus; HTN, hypertension; PDR, proliferative diabetic retinopathy; RVO, retinal vein occlusion; NA = not available; NLP, no light perception.

This study included 140 males and 93 females. The average age of these patients was 47.20 ± 20.48 years (median, 50 years; range, 3–84 years). There were 108 (46.4%) patients who had only the right eyes affected, 109 (46.8%) who had only the left eyes affected, and 16 (6.9%) who had both eyes affected; 46 (19.7%) patients had a history of diabetes mellitus (DM), and 50 (21.5%) had a history of hypertension; 16 (6.9%) patients were immunocompromised due to chronic systemic corticosteroids (*n* = 14), chronic systemic chemotherapeutic agents (*n* = 1), and allogeneic hemopoietic stem cell transplantation (*n* = 1). There were 22 (8.8%) eyes with an ocular history of glaucoma, 11 (4.4%) eyes with retinal vein occlusion, 7 (2.8%) with proliferative diabetic retinopathy, 4 (1.6%) with age-related macular degeneration, 4 (1.6%) with idiopathic choroidal neovascularization, 1 (0.4%) with corneal endothelial decompensation, 1 (0.4%) with thyroid eye disease, and 1 (0.4%) with coloboma of choroid and iris on binoculars. There were 99 (39.8%) eyes with concurrent RD at presentation, whereas 150 (60.2%) eyes did not.

The most common clinical scenario in this study was exogenous endophthalmitis (60.6%), among which endophthalmitis after perforating injuries (n = 62, 24.9%) was more common. Endogenous endophthalmitis accounted for 39.4%, and 16 patients were bilateral involvement.

Culture-positive pathogens were found in 96 (38.6%) of 249 eyes. The pathogens were divided into gram-positive cocci in 42 eyes (16.9%), fungus in 32 (12.9%) eyes, gram-negative bacilli in 14 eyes (5.6%), gram-positive bacilli in 8 patients (3.2%). Among gram-positive cocci, the most frequent pathogen was coagulase-negative staphylococci (*n* = 25, 10.0%), followed by streptococcus (*n* = 6, 2.4%) and *Staphylococcus aureus* (*n* = 6, 2.4%). The most frequent fungus was candida (*n* = 17, 6.8%). Detailed pathogens results were shown in Table 1.

99 (39.8%) eyes developed serious complications after treatment, including 53 (21.3%) eyes with retinal with/without choroid detachment, 20 (8.0%) eyes requiring evisceration or enucleation, 23 (9.2%) eyes with decreased VA to NLP, and 3 (1.2%) eyes with corneal decompensation. The mean time of follow-up was 326.18 ± 684.66 days (median, 72 days; range, 7–4,666 days).

### Clinical characteristics and treatment outcomes of endogenous and exogenous endophthalmitis

As shown in Table 2, for endogenous endophthalmitis, there were fewer males and patients with bilateral involvement mainly distributed in the endogenous entity. Patients with DM and immunosuppression were significantly more (*p* < 0.05), accounting for 29.3 and 18.3%, respectively. There was no significant difference in initial VA and RD at the initial presentation. The intervention of eyes was significantly delayed in endogenous endophthalmitis. For initial treatments, approximately 35% of eyes accepted IVI as the initial treatment in both two groups. However, more eyes (27.6%) with endogenous endophthalmitis received the nonsurgical intervention as the initial treatment when compared with the exogenous group. More than 60% of eyes needed additional treatments (61.2 and 72.8%), and approximately 40% of eyes underwent serious complications after treatment (36.7 and 41.7%). Decreased VA to NLP was more common in endogenous endophthalmitis, whereas RD was more common in exogenous endophthalmitis. There were significant differences in the distribution of pathogens between the two groups (*p* < 0.05). Exogenous endophthalmitis had a significantly better VA prognosis than endogenous endophthalmitis (*p* < 0.05), and final VA was significantly improved than initial VA (*p* < 0.001). On the other hand, there was no significant improvement after treatment for endogenous endophthalmitis (*p* = 0.281).

**TABLE 2:** Clinical characteristics and treatment outcomes of endogenous and exogenous endophthalmitis.

Number of patients and eyes	Endogenous (*n* = 98 eyes of 82 Patients)	Exogenous (*n* = 151 eyes of 151 Patients)	*p* Value
Sex(male), n(%)	39 (47.6)	101 (66.9)	***p* = 0.004**
Age, years	49.94 ± 18.60	45.71 ± 21.29	*p* = 0.153
Bilateral involvement, n(%)	16 (19.5)	0 (0)	***P<*0.001**
Presence of DM, n(%)	24 (29.3)	22 (14.6)	***p* = 0.007**
Presence of HTN, n(%)	19 (23.2)	31 (20.5)	*p* = 0.639
Immunosuppression, n(%)	15 (18.3)	1 (0.7)	***p<*0.001**
Initial logMAR BCVA	2.16 ± 0.67	2.26 ± 0.57	*p* = 0.339
RD at the initial presentation eyes(%)	45 (45.9)	54 (35.8)	*p* = 0.110
Days between onset of symptoms and operation	39.00 ± 39.24	18.83 ± 42.75	***p* = 0.005**
Initial Treatments, eyes(%)			***p<*0.001**
PPV + IVI + tamponade	18 (18.4)	32 (21.2)	
PPV + IVI without tamponade	10 (10.2)	44 (29.1)	
IVI	33 (33.7)	55 (36.4)	
Nonsurgical	27 (27.6)	17 (11.3)	
Eyes with additional treatment(%)	60 (61.2)	110 (72.8)	*p* = 0.054
Serious complications after treatment, eyes(%)	36 (36.7)	63 (41.7)	*p =* 0.748
Retinal with/without choroid detachment	11 (11.2)	42 (27.8)	
Evisceration or enucleation	8 (8.2)	12 (7.9)	
Decreased visual acuity to NLP	16 (16.3)	7 (4.6)	
Corneal decompensation	1 (1.0)	2 (1.3)	***p<*0.001**
Pathogens, eyes(%)			***p<*0.001**
Bacteria	7 (7.1)	57 (37.7)	
Fungi	24 (24.5)	8 (5.3)	
Final logMAR BCVA	2.09 ± 0.95	1.83 ± 0.84	***p =* 0.015**

BCVA, best-corrected visual acuity; DM, diabetes mellitus; HTN, hypertension; NLP, no light perception; RD, retinal detachment.

### Characteristics of endophthalmitis eyes with and without concurrent retinal detachment

**FIGURE 1:**
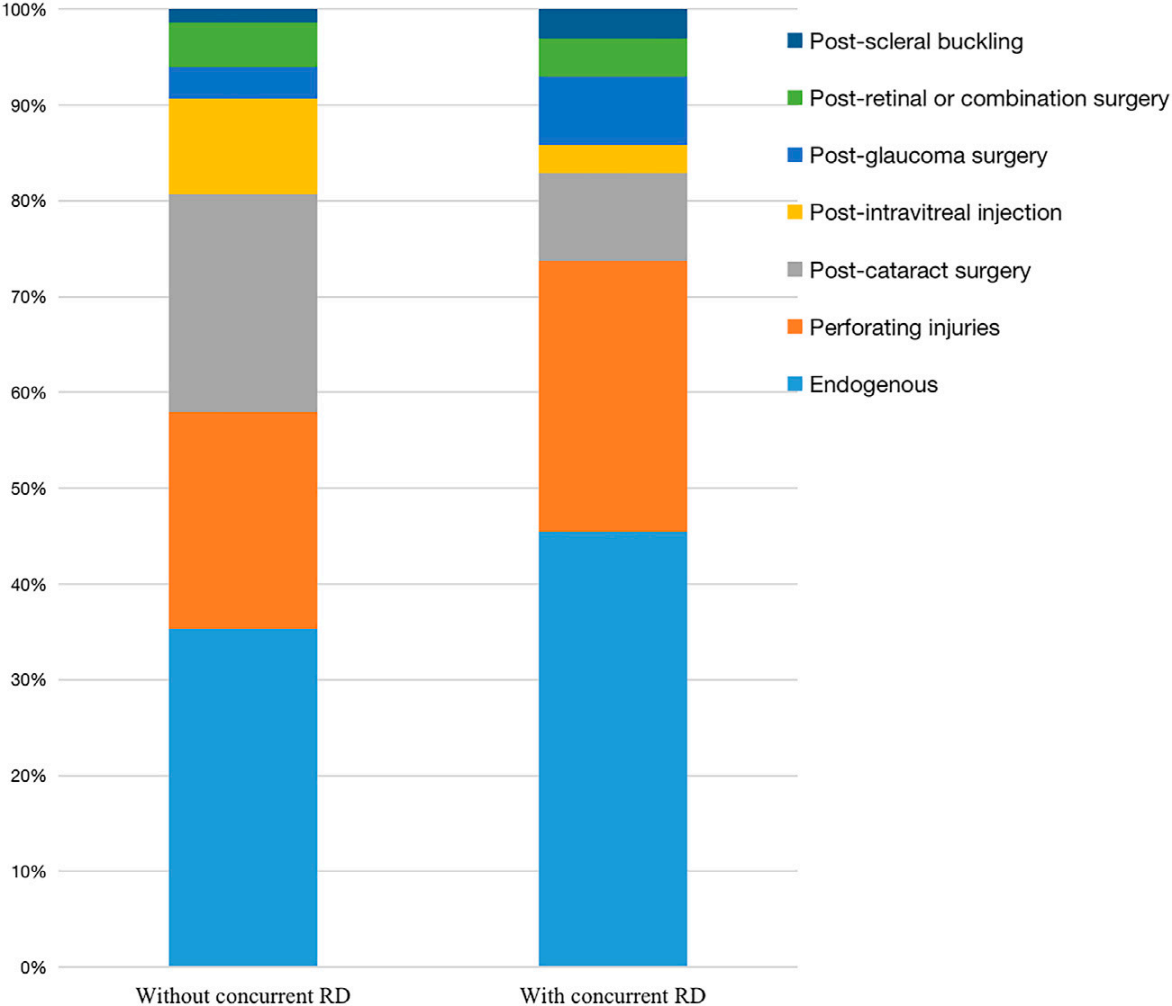
Clinical scenarios of eyes with/without concurrent retinal detachment.

The study eyes were divided into two subgroups according to whether there was concurrent RD at presentation. The clinical data were listed in Table 3. The results showed no significant difference in gender, age, presence of hypertension, presence of DM, ocular history, and initial VA between the two groups. Figure 1 shows the distribution of clinical scenarios of the two groups (*p* < 0.05).

**TABLE 3:** Clinical characteristics of endophthalmitis patients with/without concurrent retinal detachment.

Number of patients and eyes	Without concurrent RD (*n* = 150 eyes of 143 Patients)	With concurrent RD (*n* = 99 eyes of 95 Patients)	*p* Value
Sex(male), n(%)	83 (58.0)	59 (62.1)	*p* = 0.531
Age, years	49.06 ± 21.50	44.67 ± 18.26	*p* = 0.094
Side(R), eyes(%)	75 (50.0)	49 (49.5)	*p* = 0.938
Presence of DM, n(%)	25 (17.5)	23 (24.2)	*p* = 0.205
Presence of HTN, n(%)	33 (23.1)	18 (18.9)	*p* = 0.447
Immunosuppression, n(%)	9 (6.3)	7 (7.4)	*p* = 0.746
Initial logMAR BCVA	2.20 ± 0.61	2.27 ± 0.61	*p* = 0.195
Ocular history, eyes(%)	31 (21.7)	21 (22.1)	*p* = 0.938
Clinical scenarios, eyes(%)			***p* = 0.007**
Endogenous	53 (35.3)	45 (45.5)	
Perforating injuries	34 (22.7)	28 (28.3)	
Post-cataract surgery	34 (22.7)	9 (9.1)	
Post-intravitreal injection	15 (10)	3 (3.0)	
Post-glaucoma surgery	5 (3.3)	7 (7.1)	
Post-vitrectomy	7 (4.7)	4 (4.0)	
Post-scleral buckling	2 (1.3)	3 (3.0)	
Serious complications after treatment, eyes(%)	46 (30.7)	53 (53.5)	***P<*0.001**
Retinal with/without choroid detachment	26 (17.3)	27 (27.3)	
Evisceration or enucleation	11 (7.3)	9 (9.1)	
Decreased visual acuity to NLP	7 (4.7)	16 (16.2)	
Corneal decompensation	2 (1.3)	1 (1.0)	
Pathogens, eyes(%)			***p* = 0.047**
Bacteria	47 (31.3)	17 (17.2)	
Fungi	17 (11.3)	15 (15.2)	
Final logMAR BCVA	1.72 ± 0.96	2.34 ± 0.65	***p<*0.001**

BCVA, best-corrected visual acuity; DM, diabetes mellitus; HTN, hypertension; NLP, no light perception; RD, retinal detachment.

The number of eyes with serious complications after treatment in “with RD Group” was significantly more than that in “without RD group” (*p* < 0.05). For “with RD Group,” more eyes developed retinal with/without choroid detachment (27.3 vs. 17.3%) and decreased VA to NLP (16.2 vs. 4.7%). There were also differences in the distribution of pathogens between the two groups (*p* < 0.05). More pathogens of eyes without concurrent RD were bacterial (31.3 vs. 17.2%). After treatment, the final VA of eyes without RD was significantly improved from logMAR 2.20 ± 0.61 to logMAR 1.72 ± 0.96 (*p* < 0.05). However, the final VA in eyes with RD was not improved when compared with the initial values (logMAR 2.27 ± 0.61 vs. logMAR 2.34 ± 0.65).

Binary logistic regression was used to identify factors associated with final VA improvement or not, including the presence of DM, hypertension, immunosuppression, initial VA, clinical settings, pathogens, days between symptoms and operation, with/without concurrent RD at presentation, and serious complications after treatment. The results showed concurrent RD (odds ratio = 0.256, 95% confidence interval = 0.114∼0.571, *p* = 0.001) and serious complications after treatment (odds ratio = 0.191, 95% confidence interval = 0.075∼0.485, *p* = 0.001) as the factors associated with final VA (*p* < 0.05) (Supplement).

### Subgroup analysis according to different initial treatments in eyes without concurrent retinal detachment

A total of 150 eyes without concurrent RD were divided into three groups according to their initial treatment. Group 1 received PPV + IVI as initial treatment, Group 2 was initially treated with IVI only, and Group 3 was initially treated with nonsurgical treatment. The number of eyes included in the three groups was 62, 51, and 37, respectively. All patients were systematically treated with antibiotics. Males were more in Group 1 than the other two groups. There was no difference in age, presence of DM, HTN, and initial VA among the three groups. In Group 3, the days between the onset of symptoms and operation (PPV/IVI) were significantly longer than in the other two groups. Endogenous endophthalmitis was more common in Group 3, whereas in the other two groups, posttraumatic and postoperative endophthalmitis was predominant (*p* < 0.05). The proportion of bacterial infection in Group 1 and 2 was significantly higher than that in Group 3 for eyes with positive culture results (38.3%, 33.3 vs. 16.2%), whereas fungi infection was more frequent in Group 3 (*p* < 0.05) (Table 4).

**TABLE 4:** Clinical characteristics of eyes with three different initial treatments.

Category	Group 1 (*n* = 62 eyes of 61 patients)	Group 2 (*n* = 51 eyes of 49 patients)	Group 3 (*n* = 37 eyes of 34 patients)	*p* Value
Sex(male), n(%)	46 (75.4)	25 (51.0)	13 (38.2)	***p =* 0.001** ^**†**^
Age, years	50.57 ± 21.00	48.53 ± 22.69	46.82 ± 20.04	*p* = 0.408
Presence of DM, n(%)	8 (13.1)	11 (22.4)	6 (17.6)	*p* = 0.438
Presence of HTN, n(%)	12 (19.7)	12 (24.5)	9 (26.5)	*p* = 0.713
Initial logMAR BCVA	2.33 ± 0.45	2.20 ± 0.69	1.98 ± 0.67	*p* = 0.071
Days between onset of symptoms and operation	10.84 ± 15.07	7.63 ± 10.52	68.0 ± 75.72	***p<*0.001** ^**††**^
Clinical scenarios, eyes(%)				***p =* 0.006** ^**††**^
Endogenous	13 (21.0)	17 (33.3)	23 (62.2)	
Perforating injuries	16 (25.8)	12 (23.5)	6 (16.2)	
Post-cataract surgery	16 (25.8)	10 (19.6)	8 (21.6)	
Post-intravitreal injection	7 (11.3)	8 (15.7)	0	
Post-glaucoma surgery	4 (6.5)	1 (2.0)	0	
Post-vitrectomy	5 (8.1)	2 (3.9)	0	
Post-scleral buckling	1 (1.6)	1 (2.0)	0	
Pathogens, eyes(%)				***p<*0.001** ^**††**^
Bacteria	24 (38.7)	17 (33.3)	6 (16.2)	
Fungi	7 (11.3)	0 (0)	10 (27.0)	
Eyes with additional treatment(%)	40 (64.5)	44 (86.3)*	26 (70.3)	***p =* 0.029**
Eyes with additional PPV(%)	18 (29.0)	29 (56.9)	21 (56.8)	***p =* 0.003** ^**†**^
Number of total additional treatments(median)	1.52 ± 1.49 (1.0)	1.80 ± 1.18 (2.0)	2.27 ± 2.69 (2.0)	*p =* 0.256
Number of additional PPV(median)	0.40 ± 0.76 (0)	0.76 ± 0.74 (1.0)	0.78 ± 0.85 (1.0)	***p =* 0.003** ^**†**^
Number of additional IVI(median)	0.95 ± 1.28 (0)	0.92 ± 1.02 (1.0)	1.43 ± 2.26 (0)	*p =* 0.819
Serious complications after treatment, eyes(%)	25 (40.3)	14 (27.5)	7 (18.9)	*p* = 0.075
Retinal with/without choroid detachment	15 (24.2)	7 (13.7)	4 (10.8)	
Evisceration or enucleation	5 (8.1)	4 (7.8)	2 (5.4)	
Decreased visual acuity to NLP	4 (6.4)	2 (3.9)	1 (2.7)	
Corneal decompensation	1 (1.6)	1 (2.0)	0 (0)	*p* = 0.995
Final logMAR BCVA	1.77 ± 0.92	1.84 ± 0.97	1.49 ± 0.98	*p =* 0.225

*p (Bonferroni correction) < 0.05 between Groups 1 and 2.

^†^There was no difference between Group 2 and Group 3. The difference was between Group 1 and the other two groups.

^††^There was no difference between Group 1 and Group 2. The difference was between Group 3 and the other two groups.

BCVA, best-corrected visual acuity; IVI, intravitreal injection of antibiotics; NLP, no light perception; PPV, pars plana vitrectomy.

Out of 62 eyes who were initially treated with PPV + IVI, 40 (64.5%) eyes underwent additional treatments. By contrast, among eyes that received IVI as initial treatment, there were 86.3% (44 out of 51 eyes) required additional treatments, especially PPV, which were significantly different (*p* < 0.05). In Group 3, 9 patients (11 eyes) refused the further operation suggested by individual ophthalmologists. There was no difference in the number of total additional treatments and the number of additional IVI for the three groups. However, the number of additional PPV was significantly more for the initial treatment with IVI and nonsurgical treatment (*p* < 0.05) (Table 4). Although the difference was not significant, serious complications after treatment, especially retinal with/without choroid detachment, were more common in Group 1. VA improved from logMAR 2.33 ± 0.45 to logMAR 1.77 ± 0.92 (*p* < 0.05), logMAR 2.20 ± 0.69 to logMAR 1.84 ± 0.97 (*p* < 0.05), and logMAR 1.98 ± 0.67 to logMAR 1.49 ± 0.98 (*p* > 0.05), in Groups 1, 2, and 3 respectively. There were no significant differences in final logMAR BCVA among the three groups after treatment.

### Subgroup analysis according to whether silicone oil/gas tamponade was combined in Group 1

Of the 62 eyes initially treated with PPV + IVI, 17 eyes received silicone oil/gas tamponade at the same time, whereas 45 eyes did not. There was no difference in gender, age, presence of DM, HTN, and initial VA between the two groups. There are 41.2% (7 out of 17 eyes) and 73.3% (33 out of 45 eyes) requiring additional treatments (*p* < 0.05). Table 5 shows that eyes initially treated with PPV + IVI while without tamponade need more additional treatments and additional IVI (*p* < 0.05). VA improved from logMAR 2.28 ± 0.49 to logMAR 1.66 ± 0.90 (*p* < 0.05), logMAR 2.46 ± 0.30 to logMAR 2.08 ± 0.90 (*p* < 0.05) in eyes without tamponade group and with tamponade group, respectively. There were no significant differences in severe complications and final BCVA between the two ways.

**TABLE 5:** Subgroup analysis according to whether silicone oil/gas tamponade was combined in Group 1.

Category	PPV + IVI without tamponade (*n* = 45 eyes of 44 patients)	PPV + IVI with tamponade (*n* = 17 eyes of 17 patients)	*p value*
Sex(male), n(%)	32 (72.7)	14 (82.4)	*p* = 0.524
Age, years	52.11 ± 22.52	46.59 ± 15.79	*p* = 0.365
Presence of DM, n(%)	8 (18.2)	0 (0)	*p* = 0.143
Presence of HTN, n(%)	9 (20.5)	3 (17.6)	*p* = 1.0
Initial logMAR BCVA	2.28 ± 0.49	2.46 ± 0.30	*p* = 0.161
Eyes requiring additional treatment(%)	33 (73.3)	7 (41.2)	***p =* 0.018**
Eyes requiring additional IVI(%)	26 (57.8)	1 (5.9)	***p<*0.001**
Average number of total additional treatments(median)	1.76 ± 1.46 (2.0)	0.88 ± 1.41 (0.0)	***p =* 0.021**
Average number of additional PPV(median)	0.38 ± 0.58 (0)	0.47 ± 1.12 (0)	*p =* 0.433
Average number of additional IVI(median)	1.24 ± 1.33 (2.0)	0.18 ± 0.73 (0.0)	***p =* 0.001**
Serious complications after treatment, eyes(%)	17 (37.8)	8 (47.1)	*p* = 0.506
Retinal with/without choroid detachment	12 (26.7)	3 (17.6)	
Evisceration or enucleation	2 (4.4)	3 (17.6)	
Reduced visual acuity to NLP	2 (4.4)	2 (11.8)	
Corneal decompensation	1 (2.2)	0 (0)	*p* = 0.277
Final logMAR BCVA	1.66 ± 0.90	2.08 ± 0.90	*p* = 0.095

IVI, intravitreal injection of antibiotics; NLP, no light perception; PPV, pars plana vitrectomy.

## Discussion

This article described the clinical characteristics and treatment outcomes of endophthalmitis in Peking Union Medical College Hospital for the past 30 years and mainly focused on the different treatment outcomes of endophthalmitis without RD at presentation. The results showed that the most common clinical scenario was exogenous. The most frequent pathogen was gram-positive cocci, among which coagulase-negative staphylococcus was more common. After the intervention, nearly 40% of patients experienced serious posttreatment complications; the most common complication was retinal with or without choroid detachment. Concurrent RD can occur in a variety of clinical scenarios, especially endogenous and posttraumatic endophthalmitis. Patients with concurrent RD at presentation were more likely to have serious complications and poor VA after treatment; the most common one was retinal with or without choroid detachment, followed by decreased VA to NLP. The treatment of eyes without concurrent RD was studied in detail. The results showed that exogenous endophthalmitis and bacterial endophthalmitis were more likely to receive initial PPV + IVI or IVI alone, whereas endogenous endophthalmitis and fungi endophthalmitis were more likely to receive nonsurgical treatment initially. More eyes that initially received IVI alone and nonsurgical treatment required additional treatments, especially additional PPV. In addition, whether silicone oil or gas tamponade was combined with PPV affected the treatment effect outcomes. More eyes without tamponade required additional treatments, and the total number of additional treatments and the number of additional IVI were significantly higher.

Endogenous endophthalmitis was a relatively rare entity, accounting for 2–8% of endophthalmitis cases (Jackson et al., 2014). However, in this retrospective study, the incidence of endogenous endophthalmitis was 39.4%, much higher than that in other studies. We thought that the possible reason was that our hospital was a tertiary comprehensive hospital and one of the national referral centers offering health care for complex and rare disorders. There were more patients with severe systemic diseases in our hospital than in other hospitals. Therefore, more patients with endogenous endophthalmitis were included in this study. Compared with exogenous endophthalmitis, patients with endogenous endophthalmitis were more prone to binocular involvement, and there were more patients with DM or immunosuppressive diseases. Treatment was relatively delayed, causing a subset of patients to undergo nonsurgical intervention as initial treatments. The prognosis of VA was poorer, and the VA did not improve significantly after treatment. Although pathogens may vary in different clinical settings, gram-positive cocci, candida, and aspergillus were common pathogens in general (Relhan et al., 2018). Our study showed the same trend. Our study also showed that the visual and anatomical outcomes of patients with RD at presentation were worse. Concurrent RD at presentation and serious complications after treatment were significantly associated with final vision.

Initial treatment has been under discussion for a long time, especially for eyes without concurrent RD. The EVS compared immediate vitrectomy within 6 h of diagnosis with injection only. It was important to note that only core vitrectomy was performed, and posterior vitreous detachment (PVD) was avoided to prevent RD. Even so, 13% of patients who received a needle biopsy received additional treatment, compared to 8% who received a PPV as their initial treatment. At the same time, EVS found a 260% higher incidence of RD in the nonsurgical group than in the vitrectomy group, which proved that the infection itself was more devastating (Endophthalmitis Vitrectomy Study Group, 1995). In our study, fewer eyes were requiring additional treatment and the number of additional PPV was fewer for patients initially treated with PPV + IVI. A retrospective study of 104 endophthalmitis patients conducted by Kitsche M and colleagues showed that PPV + IVI as an initial treatment for endophthalmitis reduced the number of additional treatments compared with initial IVI and suggested that most eyes with endophthalmitis should receive PPV as early as possible to achieve optimal visual results (Kitsche et al., 2020). Several other retrospective studies had also shown the superiority of PPV as an initial treatment over IVI. Kuriyan AE et al. studied endophthalmitis caused by streptococcal species and showed that 69.4% of patients receiving IVI as the initial treatment required additional treatment, compared with 28.6% of patients receiving PPV + IVI as initial treatment (Kuriyan et al., 2014). Leung EH et al. and Kuhn F et al. also proposed that even if the vitreous antibiotics killed the pathogens, the residual inflammatory fragments, toxins, and various harmful enzymes could cause sustained damage to the retina. In addition, pathologies such as macular cystic edema might seriously limit its functional recovery. They considered that prolonged infection and inflammation contributed to poor visual outcomes and suggested that early rather than delayed, complete rather than partial intervention, was reasonably expected to provide further protection against retinal damage (Kuhn and Gini, 2006; Leung et al., 2016). In addition, animal experiments of pure bacterial endophthalmitis had shown that PPV + IVI could achieve faster recovery and clearer ocular media than IVI alone (McGetrick and Peyman, 1979). In our study, treatment in the nonsurgical group may be inadequate, as some patients refused to undergo further surgical treatment. Although VA improved after nonsurgical treatment, there was no statistically significant difference. The proportion of endogenous endophthalmitis and fungal infections was higher in this group, and delay in surgical treatment is likely to occur in these patients because of a poor systemic condition. However, studies by Behera UC et al., which explored whether immediate PPV altered the outcomes of fungal endophthalmitis, showed that early vitrectomy combined with intravitreal antifungal antibiotics could achieve favorable visual and structural results (Behera et al., 2018).

Outcomes such as VA and RD rates after treatment were important in assessing the benefit of early PPV. It should be noted that in our study, final VA was significantly improved in both Groups 1 and 2 compared with that before treatment. However, the final VA between the two groups was not statistically different. A multicenter study on acute postprocedure endophthalmitis of 204 eyes conducted by Soliman MK et al. evaluated the visual outcome with IVI alone and IVI + early PPV within 1 week of presentation (86.6% of patients underwent PPV on the day of diagnosis). Their results showed no difference in final VA between the two groups, and early PPV could not predict good visual outcomes (Soliman et al., 20211). Other studies such as Kuriyan AE et al., Gower EW et al., and Xu K et al. did not find an additional visual gain in early PPV (Kuriyan et al., 2014; Gower et al., 2015; Xu et al., 2018). In endophthalmitis, the reported incidence of RD after vitrectomy ranged from 5 to 21% (Foster et al., 1994). In our study, there was a nonstatistically significant trend that the incidence of severe complications after treatment was higher in the PPV + IVI group, especially the incidence of retinal with or without choroid detachment, accounting for approximately 24%. In Nelsen PT and associates’ retrospective study, 21% of patients treated with PPV + IVI had RD after treatment, compared with 9% treated with IVI alone. However, those who underwent initial vitrectomy had more severe clinical manifestations in their study; thus, the study could not determine whether the difference was significant (Nelsen et al., 1985). However, in some studies such as EVS and Soliman MK et al., there was no difference in RD rates between the two initial treatment modalities (Endophthalmitis Vitrectomy Study Group, 1995; Soliman et al., 20211). No cases of RD were reported in a study of 47 endophthalmitis patients by Kuhn F et al. The authors attributed its favorable anatomical success to more thorough vitreous removal compared to EVS (Kuhn and Gini, 2005). Differences among various studies were difficult to compare due to time, etiology, the composition of pathogens, etc. In our study, we believed that a possible reason was that in clinical practice, ophthalmologists always preferred PPV for more severe endophthalmitis (such as more vitreous opacity, more rapid progress, poorer red reflex, etc.), which meant that there was a selection bias in the choice of treatment. In other words, patients in the initial PPV group may have more severe clinical manifestations before treatment, which caused the visual and anatomical results in our study. As for the mechanism of RD after PPV, there were several possible reasons as follows. First, severe inflammation induced by endophthalmitis may cause a large number of sequelae to the vitreous and retina, making the eyes prone to RD. Second, undiluted vitreous sampling at the beginning of PPV surgery could lead to lower intraocular pressure and the potential risk of vitreoretinal traction and choroid detachment or bleeding. Retinal tears and iatrogenic holes may occur during surgery. In addition, without a vitreous gel cushion after vitrectomy, the jet stream effect was produced by rapid injection of antibiotics through a small needle, resulting in retinal injury (Nelsen et al., 1985; Machemer and Norton, 1968; Chiquet et al., 2016; Dong et al., 20211).

Vitro experiments had shown that silicone oil can reduce the proliferation of bacteria. In addition, silicone oil was highly hydrophobic and had high interfacial tension, which could limit the movement of pathogens and reduce their conductivity. Vitrectomy with silicone oil had the tamponade effect on the retina and could reduce the incidence of RD (Okhravi et al., 1997; Ozdamar et al., 1999). In a rabbit model of posttraumatic endophthalmitis, Mansour AM and colleagues found that eyes with fluid–gas exchange had less intraocular inflammation than that without gas filling, and they suggested that vitreous gas might be effective on endophthalmitis (Mansour et al., 1991). In our study, patients with silicone oil or gas tamponade required fewer additional treatments, especially additional IVI. For severe complications, although the difference was not statistically significant, the retinal with/without choroid detachment was less, whereas evisceration or enucleation and reduced VA to NLP were slightly more. Therefore, the use of silicone oil or gas tamponade in vitrectomy therapy without concurrent RD may help control the infection process and reduce the risk of RD. However, the advantages of bacteriostasis and tamponade should be carefully weighed against the risk of silicone oil toxicity to the retina and the altered vitreous antibiotic concentration (Tode et al., 2016).

Some limitations still existed. This was a retrospective study, and some bias in treatment selection cannot be ignored. Further prospective and randomized studies may be needed to address treatment selection in more detail. Second, different surgeons may affect surgical outcomes. In addition, various duration of follow-up may have an impact on the final vision.

## Conclusion

Endophthalmitis is a devastating intraocular disease and needs early intervention. Endogenous endophthalmitis has a poorer visual prognosis than exogenous entity. Eyes with concurrent RD at presentation usually underwent worse visual and anatomical outcomes. PPV + IVI as an initial treatment may reduce the number of additional therapies.

## Data Availability

The raw data supporting the conclusion of this article will be made available by the authors, without undue reservation.

## Abbreviations

Anti-VEGF, antivascular endothelial growth factor; BCVA, best-corrected visual acuity; DM, diabetes mellitus; EVS, Endophthalmitis Vitrectomy Study; IVI, intravitreal injection of antibiotics; LogMAR, the logarithm of the minimum angle of resolution; LP, light perception; NLP, no light perception; RD, retinal detachment; PPV, pars plana vitrectomy; PVD, posterior vitreous detachment.

## References

[B1] Behera U. C. Budhwani M. Das T. Basu S. Padhi T. R. Barik M. R. (2018). Role of early vitrectomy in the treatment of fungal endophthalmitis. Retina 38 (7), 1385–1392. 10.1097/IAE.0000000000001727 28541964

[B2] Chiquet C. Aptel F. Combey-de Lambert A. Bron A. M. Campolmi N. Palombi K. (2016). Occurrence and risk factors for retinal detachment after pars plana vitrectomy in acute postcataract bacterial endophthalmitis. Br. J. Ophthalmol. 100 (10), 1388–1392. 10.1136/bjophthalmol-2015-307359 26802175

[B3] Clarke B. Williamson T. H. Gini G. Gupta B. (2018). Management of bacterial postoperative endophthalmitis and the role of vitrectomy. Surv. Ophthalmol. 63 (5), 677–693. 10.1016/j.survophthal.2018.02.003 29453989

[B4] Dong L. K. Shields R. A. Subramanian S. Lee R. Wa C. A. Ruby A. J. (2021). Features and outcomes of eyes that underwent surgical repair of rhegmatogenous retinal detachments after being treated for acute endophthalmitis. Retina 41 (8), 1612–1617. 10.1097/IAE.0000000000003091 33394997

[B5] Durand M. L. (2017). Bacterial and fungal endophthalmitis. Clin. Microbiol. Rev. 30 (3), 597–613. 10.1128/CMR.00113-16 28356323 PMC5475221

[B6] Durand M. L. (2013). Endophthalmitis. Clin. Microbiol. Infect. 19 (3), 227–234. 10.1111/1469-0691.12118 23438028 PMC3638360

[B7] Endophthalmitis Vitrectomy Study Group (1995). Results of the Endophthalmitis Vitrectomy Study. A randomized trial of immediate vitrectomy and of intravenous antibiotics for the treatment of postoperative bacterial endophthalmitis. Arch. Ophthalmol. 113 (12), 1479–1496. 7487614

[B8] Foster R. E. Rubsamen P. E. Joondeph B. C. Flynn H. W. Jr Smiddy W. S. (1994). Concurrent endophthalmitis and retinal detachment. Ophthalmology 101 (3), 490–498. 10.1016/s0161-6420(94)31308-x 8127569

[B9] Gower E. W. Keay L. J. Stare D. E. Arora P. Cassard S. D. Behrens A. (2015). Characteristics of endophthalmitis after cataract surgery in the United States medicare population. Ophthalmology 122 (8), 1625–1632. 10.1016/j.ophtha.2015.04.036 26045364 PMC4516609

[B10] Jackson T. L. Paraskevopoulos T. Georgalas I. (2014). Systematic review of 342 cases of endogenous bacterial endophthalmitis. Surv. Ophthalmol. 59 (6), 627–635. 10.1016/j.survophthal.2014.06.002 25113611

[B11] Kitsche M. Herber R. Pillunat L. E. Terai N. (2020). Clinical and visual outcome of endophthalmitis patients: A single-center experience. Graefes Arch. Clin. Exp. Ophthalmol. 258 (1), 183–189. 10.1007/s00417-019-04480-2 31637487

[B12] Kuhn F. Gini G. (2005). Ten years after. are findings of the Endophthalmitis Vitrectomy Study still relevant today? Graefes Arch. Clin. Exp. Ophthalmol. 243 (12), 1197–1199. 10.1007/s00417-005-0082-8 16136321

[B13] Kuhn F. Gini G. (2006). Vitrectomy for endophthalmitis. Ophthalmology 113 (4), 714. 10.1016/j.ophtha.2006.01.009 16581433

[B14] Kuriyan A. E. Weiss K. D. Flynn H. W. Jr Smiddy W. E. Berrocal A. M. Albini T. A. (2014). Endophthalmitis caused by streptococcal species: Clinical settings, microbiology, management, and outcomes. Am. J. Ophthalmol. 157 (4), 774–780. 10.1016/j.ajo.2013.12.026 24418264 PMC3972252

[B15] Leung E. H. Kuriyan A. E. Flynn H. W. Jr Miller D. Huang L. C. (2016). Persistently vitreous culture-positive exogenous bacterial endophthalmitis. Am. J. Ophthalmol. 165, 16–22. 10.1016/j.ajo.2016.02.017 26921804 PMC4870093

[B16] Machemer R. Norton E. W. (1968). Experimental retinal detachment in the owl monkey. I. Methods of producation and clinical picture. Am. J. Ophthalmol. 66 (3), 388–396. 10.1016/0002-9394(68)91522-5 4970985

[B17] Maguire J. I. (2008). Postoperative endophthalmitis: Optimal management and the role and timing of vitrectomy surgery. Eye (Lond) 22 (10), 1290–1300. 10.1038/eye.2008.51 18356929

[B18] Mansour A. M. Ferguson E. 3rd Lucia H. Rajashekhar M. Li H. Margo T. (1991). Vitreous replacement by gas as a therapeutic modality in bacterial endophthalmitis. Graefes Arch. Clin. Exp. Ophthalmol. 229 (5), 468–472. 10.1007/BF00166312 1937081

[B19] McGetrick J. J. Peyman G. A. (1979). Vitrectomy in experimental endophthalmitis: Part II--Bacterial endophthalmitis. Ophthalmic Surg. 10 (3), 87–92. 460811

[B20] Nelsen P. T. Marcus D. A. Bovino J. A. (1985). Retinal detachment following endophthalmitis. Ophthalmology 92 (8), 1112–1117. 10.1016/s0161-6420(85)33916-7 3876533

[B21] Okhravi N. Towler H. M. Hykin P. Matheson M. Lightman S. (1997). Assessment of a standard treatment protocol on visual outcome following presumed bacterial endophthalmitis. Br. J. Ophthalmol. 81 (9), 719–725. 10.1136/bjo.81.9.719 9422921 PMC1722324

[B22] Ozdamar A. Aras C. Ozturk R. Akin E. Karacorlu M. Ercikan C. (1999). *In vitro* antimicrobial activity of silicone oil against endophthalmitis-causing agents. Retina 19 (2), 122–126. 10.1097/00006982-199902000-00006 10213237

[B23] Relhan N. Forster R. K. Flynn H. W. Jr (2018). Endophthalmitis: Then and now. Am. J. Ophthalmol. 187, xx–xxvii. 10.1016/j.ajo.2017.11.021 29217351 PMC5873969

[B24] Simunovic M. P. Rush R. B. Hunyor A. P. Chang A. A. (2012). Endophthalmitis following intravitreal injection versus endophthalmitis following cataract surgery: Clinical features, causative organisms and post-treatment outcomes. Br. J. Ophthalmol. 96 (6), 862–866. 10.1136/bjophthalmol-2011-301439 22446145

[B25] Soliman M. K. Gini G. Kuhn F. Parolini B. Ozdek S. Adelman R. A. (2021). Visual outcome of early vitrectomy and intravitreal antibiotics in acute postsurgical and postintravitreal injection endophthalmitis: European vitreo-retinal society endophthalmitis study report two. Retina 41 (2), 423–430. 10.1097/IAE.0000000000002856 32467482

[B26] Spelta S. Di Zazzo A. Antonini M. Bonini S. Coassin M. (2021). Does endogenous endophthalmitis need a more aggressive treatment? Ocul. Immunol. Inflamm. 29 (5), 937–943. 10.1080/09273948.2019.1705497 31951759

[B27] Tode J. Purtskhvanidze K. Oppermann T. Hillenkamp J. Treumer F. Roider J. (2016). Vision loss under silicone oil tamponade. Graefes Arch. Clin. Exp. Ophthalmol. 254 (8), 1465–1471. 10.1007/s00417-016-3405-z 27278374

[B28] Xu K. Chin E. K. Bennett S. R. Williams D. F. Ryan E. H. Dev S. (2018). Endophthalmitis after intravitreal injection of vascular endothelial growth factor inhibitors: Management and visual outcomes. Ophthalmology 125 (8), 1279–1286. 10.1016/j.ophtha.2018.01.022 29477689

