# Supplementary material for: Endophthalmitis at a tertiary referral center: Characteristics and treatment outcomes over three decades
Source: Front Cell Dev Biol. 2022 Aug 8;10:952375. doi: 10.3389/fcell.2022.952375 (PMC9395169; doi:10.3389/fcell.2022.952375)
Supplement: Supplementary file 1 [file Table1.DOCX]

**Supplement.** Possible factors associated with final VA improvement or not.

| **Factors** | **OR** | **95% CI** | ***P* value** |
| --- | --- | --- | --- |
| **Presence of DM** | 1.51 | 0.528-4.326 | *P*=0.441 |
| **Presence of HTN** | 0.610 | 0.219-1.700 | *P*=0.345 |
| **Immunosuppression** | 0.468 | 0.075-2.911 | *P*=0.416 |
| **Initial logMAR BCVA** | 1.365 | 0.686-2.714 | *P*=0.375 |
| **RD at the initial presentation eyes** | 0.256 | 0.114-0.571 | ***P*=0.001** |
| **Days between onset of symptoms** **and operation** | 1.002 | 0.994-1.011 | *P*=0.633 |
| **Clinical settings** |  |  | *P*=0.781 |
| **Pathogens** |  |  | *P*=0.939 |
| **Serious complications after treatment** | 0.191 | 0.075-0.485 | ***P*=0.001** |

BCVA=best corrected visual acuity, DM=diabetes mellitus, HTN=hypertension, RD=retinal detachment, OR= odds ratio, CI=confidence interval.
